# The physiological foundations of critical care medicine: the contribution of Joseph Milic-Emili, a physiologist “by hook or by crook”

**DOI:** 10.1186/s13054-022-03919-z

**Published:** 2022-02-08

**Authors:** V. Marco Ranieri, Claude Guérin

**Affiliations:** 1 Department of Emergency and Intensive Care Medicine, Alma Mater Studiorum University of Bologna, IRCCS Policlinico di Sant’Orsola, Bologna, Italy; 2 Service de Médecine Intensive-Réanimation, Hôpital Edouard Herriot, 5 Place d’Arsonval, 69003 Lyon, France; 3 Université de Lyon, Lyon, France; 4 Institut Mondor de Recherche Biomédicales INSERM 955, Créteil, France

The profound understanding of the physiology of organ failure and of the supportive/replacement care of failing organs is the cornerstone that identifies the clinical and scientific uniqueness of critical care medicine. The current SARS-CoV-2 pandemic reminds us of this foundation on a daily basis. Optimal management of COVID-19 is based on an understanding of the mechanisms of acute respiratory failure, an understanding of the effect of the virus on the respiratory system, and of the lung involvement with respect to the pulmonary micro-circulation. This understanding comes from good, precise, adequately interpreted measurements. After years of evidence-based medicine, which has its own merit, the modern era of managing critically ill patients highlights the need for precision and individualized medicine.

Joseph Milic-Emili (“Milic” to his friends and colleagues) passed away on January 8, 2022 (https://www.meakinsmcgill.com). Milic obtained his medical degree in 1955 at the University of Milan and started his career in the department of physiology working with Rodolfo Margaria. There, together with Jean-Marie Petit, he provided the first description of the methods to obtain the “optimal” measurement of esophageal pressure as an index of pleural pressure using a rubber balloon [1]. In 1957 Milic moved to Liege where he developed the esophageal balloon-catheter method to determine the electrical activity of the diaphragm [2]. In 1960 he moved to Harvard University, where he produced one of the first studies on the partitioning of respiratory mechanics between lung and chest wall [3]. In 1963 he joined McGill University where he started to work on the quantitative description of factors governing distribution of gas within the lung and in 1966 he published a study describing that: (a) the alveoli in the upper lung regions are more expanded than those in the lower lung zones; (b) above the functional residual capacity (FRC), relationship between regional and overall lung volume is linear, but the ventilation per alveolus is higher in the lower lung zones; and, (c) below the FRC, there is a progressive small airway closure in the dependent lung zones as residual volume is approached until a minimal limiting volume (closing volume) is reached [4]. Later, he was the first to describe a simple and non-invasive technique to assess the control of breathing (the mouth occlusion method) [5].

From 1979 to 1994, Milic served as Director of the Meakins-Christie Labs, a research center founded in 1972 by Dr. Peter Macklem that since its inception, has established itself as an institution dedicated to the study of all respiratory disorders and served as an “incubator” for the new generations of basic, translational and clinical researchers on respiratory diseases. During his tenure, Milic sensed the potential, scientific, and clinical development that could be achieved by applying results of his research as a physiologist "by hook or by crook" [6] to the context of the different forms of acute respiratory failure and mechanical ventilation. In this perspective, he “invaded” the field of critical care medicine and worked with, taught, and educated several generations of clinicians with an interest in applied physiology.

Giving a clinical prospective to his basic physiological studies [1–5], Milic provided the foundations of some of the clinical, technological, and research in the field of acute respiratory failure and mechanical ventilation. First, Milic original work on esophageal pressure [1, 3] was translated and validated as a clinical procedure [7] that allowed examination of the role of intrinsic positive end-expiratory pressure and inspiratory muscle effort in COPD patients [8] and examination of the role of chest wall mechanics in critically ill patients [9]. This led to the clinical use of measurements of esophageal pressure to partition respiratory system mechanics between lung and chest wall in critically ill patients to the point that recent studies suggest that minimizing the pressure applied to the lung (i.e., transpulmonary pressure) may decrease mortality [10]. Second, assessment of the electrical activity of the diaphragm through an esophageal catheter [2] is a monitoring option and can be used to drive ventilatory support [11]. Third, the quantitative description of the regional distribution of gas within the lung [4] guided analysis of the volume-pressure relationship to assess the effects of PEEP in terms of alveolar recruitment/overinflation [12] inspiring the concept of using driving pressure as a monitoring tool for ventilator-induced lung injury [13]. Fourth, P_0.1_ [5] is now a simple and noninvasive measurement of respiratory drive that is available on almost all ventilators and has gained increasing interest with the recognition that ventilator-induced lung injury can occur during assisted breathing [14].

Death itself is an inevitable event, even for great scientists. Therefore, the fact that Milic has left his family, his friends, his colleagues does not in itself justify public recognition through publication of this article. We, his students, his colleagues, his friends do not just propose to the readers of the journal a tribute to the scientific and human figure of Milic. Instead, we wrote these words to remind all of us of the importance of recognizing and enhancing the scientific foundations of our discipline. We come from afar and we go far. The contextualization of what we learned from Milic, of the rigorous methods he implemented in the physiology lab first and in the clinical setting later will allow all of us to advance the scientific development of our discipline and most importantly to provide better care for critically ill patients. Thank you Milic.

## Data Availability

Not applicable.

## References

[1] Petit JM, Milic-Emili G (1958). Measurement of endoesophageal pressure. J Appl Physiol.

[2] Petit JM, Milic-Emili G, Delhez L (1960). Role of the diaphragm in breathing in conscious normal man: an electromyographic study. J Appl Physiol.

[3] Milic-Emili J, Mead J, Turner JM, Glauser EM (1964). Improved technique for estimating pleural pressure from esophageal balloons. J Appl Physiol.

[4] Milic-Emili J, Henderson JA, Dolovich MB, Trop D, Kaneko K (1966). Regional distribution of inspired gas in the lung. J Appl Physiol.

[5] Whitelaw WA, Derenne JP, Milic-Emili J (1975). Occlusion pressure as a measure of respiratory center output in conscious man. Respir Physiol.

[6] Milic-Emili J (2003). A respiratory physiologist by hook or by crook. Am J Respir Crit Care Med.

[7] Baydur A, Behrakis PK, Zin WA, Jaeger M, Milic-Emili J (1982). A simple method for assessing the validity of the esophageal balloon technique. Am Rev Respir Dis.

[8] Petrof BJ, Legare M, Goldberg P, Milic-Emili J, Gottfried SB (1990). Continuous positive airway pressure reduces work of breathing and dyspnea during weaning from mechanical ventilation in severe chronic obstructive pulmonary disease. Am Rev Respir Dis.

[9] Guerin C, Coussa ML, Eissa NT, Corbeil C, Chasse M, Braidy J, Matar N, Milic-Emili J. Lung and chest wall mechanics in mechanically ventilated COPD patients. J Appl Physiol 1993;74(4):1570–1580. 10.1152/jappl.1993.74.4.1570 8514671

[10] Sarge T, Baedorf-Kassis E, Banner-Goodspeed V, Novack V, Loring SH, Gong MN, Cook D, Talmor D, Beitler JR (2021). Group EP-S: effect of esophageal pressure-guided positive end-expiratory pressure on survival from acute respiratory distress syndrome: a risk-based and mechanistic reanalysis of the EPVent-2 trial. Am J Respir Crit Care Med.

[11] Sinderby C, Navalesi P, Beck J, Skrobik Y, Comtois N, Friberg S, Gottfried SB, Lindstrom L (1999). Neural control of mechanical ventilation in respiratory failure. Nat Med.

[12] Ranieri VM, Eissa NT, Corbeil C, Chasse M, Braidy J, Matar N, Milic-Emili J (1991). Effects of positive end-expiratory pressure on alveolar recruitment and gas exchange in patients with the adult respiratory distress syndrome. Am Rev Respir Dis.

[13] Amato MB, Meade MO, Slutsky AS, Brochard L, Costa EL, Schoenfeld DA, Stewart TE, Briel M, Talmor D, Mercat A (2015). Driving pressure and survival in the acute respiratory distress syndrome. N Engl J Med.

[14] Brochard L, Slutsky A, Pesenti A (2017). Mechanical ventilation to minimize progression of lung injury in acute respiratory failure. Am J Respir Crit Care Med.

